# Feruloyl esterase immobilization in mesoporous silica particles and characterization in hydrolysis and transesterification

**DOI:** 10.1186/s12858-018-0091-y

**Published:** 2018-02-02

**Authors:** Cyrielle Bonzom, Laura Schild, Hanna Gustafsson, Lisbeth Olsson

**Affiliations:** 1 Department of Biology and Biological Engineering, Industrial Biotechnology Division, Chalmers University of Technology, SE-412 96 Gothenburg, Sweden; 2 Department of Chemical and Biological Engineering, Applied Surface Chemistry Division, Chalmers University of Technology, SE-412 96 Gothenburg, Sweden; 3 Department of Applied Physics, Biological Physics Division, Chalmers University of Technology, SE-412 96 Gothenburg, Sweden

**Keywords:** Kinetic parameters, Feruloyl esterase selectivity, Enzyme reusability, E-FAERU, Enzyme stability, Mesoporous silica

## Abstract

**Background:**

Enzymes display high reactivity and selectivity under natural conditions, but may suffer from decreased efficiency in industrial applications. A strategy to address this limitation is to immobilize the enzyme. Mesoporous silica materials offer unique properties as an immobilization support, such as high surface area and tunable pore size.

**Results:**

The performance of a commercially available feruloyl esterase, E-FAERU, immobilized on mesoporous silica by physical adsorption was evaluated for its transesterification ability. We optimized the immobilization conditions by varying the support pore size, the immobilization buffer and its pH. Maximum loading and maximum activity were achieved at different pHs (4.0 and 6.0 respectively). Selectivity, shown by the transesterification/hydrolysis products molar ratio, varied more than 3-fold depending on the reaction buffer used and its pH. Under all conditions studied, hydrolysis was the dominant activity of the enzyme. pH and water content had the greatest influence on the enzyme selectivity and activity. Determined kinetic parameters of the enzyme were obtained and showed that K_m_ was not affected by the immobilization but k_cat_ was reduced 10-fold when comparing the free and immobilized enzymes. Thermal and pH stabilities as well as the reusability were investigated. The immobilized biocatalyst retained more than 20% of its activity after ten cycles of transesterification reaction.

**Conclusions:**

These results indicate that this enzyme is more suited for hydrolysis reactions than transesterification despite good reusability. Furthermore, it was found that the immobilization conditions are crucial for optimal enzyme activity as they can alter the enzyme performance.

**Electronic supplementary material:**

The online version of this article (10.1186/s12858-018-0091-y) contains supplementary material, which is available to authorized users.

## Background

Although enzymes exhibit high reactivity and selectivity in their natural environment, they may suffer from denaturation under reaction conditions that differ from their natural ones, thus restricting their industrial use. One strategy used to solve this problem is to immobilize the enzymes [1]. Immobilized enzymes can exhibit enhanced properties in terms of activity, specificity or selectivity. The proposed mechanisms behind those apparent alterations have been reviewed recently [2]. Enzymes can be immobilized using different techniques such as encapsulation, enzyme cross linking or immobilization on a solid support by physical adsorption or covalent linkages which have been reviewed recently [3]. In this study, immobilization relied on physical adsorption.

Materials made of mesoporous silica (MPS) have recently become interesting as support materials for immobilized enzymes. MPS offers unique properties such as high surface area and tunable pore size over the range 2–50 nm [4]. They also have high chemical and mechanical stability [5]. Among the variety of existing MPS materials, SBA-15 (Santa Barbara Amorphous Type 15) present some interesting properties. SBA-15 has a well ordered network of hexagonal silica structures [6] of which the pore size and thickness of the walls can be tuned by varying the synthesis conditions. Both pore size and wall thickness are uniform throughout the material. Different kinds of enzymes, such as feruloyl esterases, lipases, glucose oxidases and papain, have been successfully immobilized in this type of mesoporous support [7–9]. It has also been shown that immobilization can increase the enzyme stability, increasing its half-life by more than 60,000 times in some cases [10]. Furthermore, enzyme immobilization can modify the specific activity of the enzyme or even change its selectivity and specificity in synthetic reactions, almost doubling the final product yield [2, 7, 11]. These findings are promising, and indicate that it could be possible to tune the activity of an enzyme simply by immobilizing it in a particular way. Moreover, SBA-15 can provide a sheltered environment for the enzymes, as is the case with other porous materials. It has been suggested that the porous network in which the enzymes are immobilized can create concentration gradients (pH, solvent, substrate, etc.), thereby having a positive effect on the biocatalyst [2]. The properties of MPS thus make it an attractive immobilization support.

Feruloyl esterases (FAEs), also known as ferulic acid esterases, cinnamoyl esterases or cinnamoyl ester hydrolases, are enzymes belonging to a subclass of carboxylic ester hydrolases (E.C. 3.1.1.73). In nature, these enzymes catalyze the hydrolysis of ester linkages, releasing ferulic acid (FA) and other hydroxycinnamic acids from plant cell wall material. FAEs are of particular importance in the degradation of plant cell walls where they have been shown to act synergistically with other carbohydrate-degrading enzymes [12]. FAEs have found applications in many industrial sectors such as the pulp and paper industry, bioethanol production and the feed industry [13].

Other sectors in which applications are found for FAEs are the cosmetic and pharmaceutical industries [13], as FAEs are able to release FA and other hydroxycinnamic acids, which have been reported to have antitumor, antimicrobial and/or antioxidant effects [14]. FAEs can also catalyze the synthesis of various hydroxycinnamic acids through esterification and transesterification reactions [15, 16]. As with other synthetic reactions, transesterification reactions are more likely to occur when the water content of the reaction medium is low. Reducing the water content decreases the water activity in the reaction system, thereby promoting synthetic reactions, and allowing hydrolytic enzymes to act as biosynthetic tools [7, 17–19]. Being able to synthesize and tune the properties of hydroxycinnamic acids through transesterification would be valuable in expanding their applicability. Modification of hydroxycinnamic acids to alter their solubility or hydrophobicity, may be necessary prior to their use in cosmetic or pharmaceutical products, for formulation purposes. These modifications are difficult using traditional chemistry [13]. The development of enzymatic synthesis tools is thus both attractive and promising.

The objective of this study was to gain insight into how the immobilization of a commercially available FAE, E-FAERU, derived from a rumen microorganism, in MPS by physical adsorption affects the enzyme and its selectivity. The substrates used in the reactions were methyl ferulate (MFA) and 1-butanol. Parameters such as pH, buffer and water content were varied during immobilization to assess their impact on immobilization efficiency and on enzyme selectivity. Another aim of the study was to evaluate the effects of immobilization on the kinetic parameters of the FAE. We first identified the optimum reaction conditions, and then determined the kinetic parameters K_m_ and k_cat_ using the model substrate MFA. Finally, the industrial potential of the enzyme was investigated by evaluating its stability and reusability.

## Methods

### Chemicals and enzyme

Ferulic acid and methyl ferulate were purchased from Apin Chemicals Ltd. (Abingdon, UK). 1-Butanol, methanol, glacial acetic acid, sodium carbonate and bis(2-hydroxyethyl)amino-tris(hydroxymethyl)methane (Bis-Tris) were purchased from Sigma-Aldrich (St Louis, MO, USA). 3-(N-morpholino)propanesulfonic acid (MOPS) was purchased from Amresco Inc. (Cleveland, OH, USA). Butyl ferulate (BFA) was kindly provided by Evangelos Topakas (National Technical University of Athens, Greece). The enzyme, E-FAERU, a feruloyl esterase from a rumen microorganism was purchased from Megazyme as a monocomponent enzyme (single band on an SDS-PAGE gel) (Bray, Co. Wicklow, Ireland).

### Mesoporous silica support

SBA-15 was used as the MPS support. SBA-15 MPS with three different pore sizes were synthesized using protocols adapted from Zhao et al. [6] Pluronic P123 is used as structure directing agent and TEOS as the silica source. 4.0 g of P123 was dissolved in 120 g of 2 M HCl and 30 g of deionized water and the mixture was vigorously stirred for 2 h at 35 °C. 8.5 g of TEOS was added and the solution was stirred at 35 °C for an additional 24 h. The gel mixture was transferred to stainless steel pressure autoclaves with Teflon containers and was aged for 24 h at 80, 120 or 140 °C, depending on the desired pore diameter. The solid precipitate was recovered by vacuum filtration, washed with deionized water and dried. Finally, the template was removed from the product through calcination, by increasing the temperature from room temperature to 500 °C during 8 h followed by heating at 500 °C for another 6 h. The samples were then characterized as described by Gustafsson et al. [20]. The main particle properties are summarized in Table 1 and a TEM image, representative of the different obtained materials and showing their hexagonal pore structure, is presented in the supplementary material (Additional file 1 Figure S1).

**Table 1:** Properties of the MPS materials used for enzyme immobilization

Pore diameter (nm)	BET surface area (m^2^·g^− 1^)	Specific pore volume (cm^3^·g^− 1^)
10.1	439	1.11
9.9	433	1.11
7.8	679	1.17
5.0	924	0.73

### Immobilization of the enzyme

The immobilization procedure was adapted from Thörn et al. [7]. Briefly, the enzyme solution was spin-filtered (Amicon Ultra–0.5 mL 10 K ultracel membrane, Millipore, Billerica, Massachusetts, USA) with the same buffers as those used during immobilization (0.2 M phosphate-citrate in the pH range 4.0–8.0, or 0.1 M MOPS in the pH range 6.0–7.5, or 0.1 M Bis-Tris in the pH range 6.0–7.5). The MPS was washed with immobilization buffer to remove possible residues from material preparation. To evaluate the effect of the immobilization buffer, the enzyme solution was mixed with 2 mg MPS (0.2 mg_enz_ ⋅ mL_buffer_^− 1^ and 44 mL_buffer_ ⋅ g_MPS_^− 1^). For activity assays, the enzyme solution was mixed with 20 mg washed MPS (0.4 mg_enz_ ⋅ mL_buffer_^− 1^ and 44 mL_buffer_ ⋅ g_MPS_^− 1^). Then left on a rotating wheel (Rotator SB3, Stuart, Stone, Staffordshire, UK) overnight at 40 rpm and room temperature (RT) in a micro-centrifuge tube. Immobilization was stopped by centrifugation (5 min, 15,000 × g, RT) and the MPS carrying enzymes was washed 3 times with the immobilization buffer to remove unbound enzyme. The MPS was then dried in a vacuum concentrator (RVC 2–18, Christ, Osterode am Harz, Germany) to remove residual water before activity tests were performed.

### Loading experiments

Loading experiments were performed according to the protocol described previously by varying the enzyme concentration from 0.1 to 1 mg_enz_ ⋅ mL_buffer_^− 1^. The chosen buffer for the experiments was 0.2 M phosphate-citrate pH 6.5. The experiments were done in triplicates.

### Determination of adsorption yields and enzyme loading

The amount of immobilized enzyme was calculated by measuring the amount of residual enzyme in the supernatants after the immobilization process, and in the subsequent washing buffer, using the Biorad DC protein assay kit with bovine serum albumin (BSA) as a standard (Biorad, Hercules, California, USA). The enzyme loading was then calculated as the quantity of adsorbed enzyme per mg MPS.

### Enzymatic assays

**Fig. 1:**
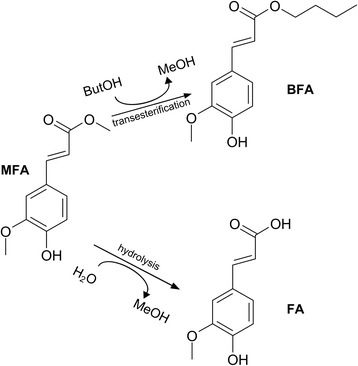
Reaction scheme of feruloyl esterase catalysis in a buffer-butanol mixture where both transesterification reaction converting methyl ferulate (MFA) to butyl ferulate (BFA) and hydrolysis reaction converting MFA to ferulic acid (FA) take place

All enzymatic assays were performed using MFA as the substrate. In the buffer-butanol mixture used during the transesterification reactions, MFA can be converted to either FA via hydrolysis or BFA via transesterification (see reaction scheme in Fig. 1). Two different types of assay were performed. A spectrophotometric assay when hydrolysis was studied, allowing the detection of MFA and FA; An HPLC measurement when studying transesterification allowing the detection of MFA, FA and BFA. All assays were performed in triplicate. For hydrolysis experiments, corresponding triplicate blanks were made using buffer (the same buffer and pH as in the reaction) instead of the enzyme and the activity of the enzyme was corrected by the background. The transesterification reaction was performed without adding enzyme and monitored during 48 h. Results showed no difference whether or not MPS were added to the reaction mixture with no detectable conversion of MFA into BFA or FA. Moreover, the MFA concentration remained the same during the time course of the experiment. Consequently, MPS did not as such contribute to the conversion of MFA and addition of enzyme greatly accelerated the reaction towards its thermodynamic equilibrium.

### Hydrolysis reactions

Hydrolysis reactions were performed in buffer using a 0.5 mM MFA stock solution. MFA was prepared by dissolving the powder first in methanol (5% (*v*/v)) and then in the buffer (95% (v/v)). For the free enzyme, 900 μL of the substrate stock solution was pre-incubated at 40 °C, and the reaction was started by the addition of 300 μL of 5000-fold diluted enzyme in the chosen buffer. For the immobilized enzyme, 0.5 mg MPS carrying the enzyme was resuspended in 300 μL buffer. The reaction was started by adding 900 μL preheated buffer containing the substrate. The first sample, 100 μL, was taken immediately, and samples were then taken every minute for 10 min. Samples were immediately quenched by the addition of 110 μL 1 M sodium carbonate. MPS particles were separated from the sample by centrifugation (15,000 × g, 5 min, 4 °C). MFA and FA were quantified using 200 μL quenched samples in micro-titer plates, by spectrophotometry; the absorbance being measured at 340 nm. Standard curves were obtained for MFA and FA for each set of experimental conditions to allow quantification. The activity at *t* = 10 min was then calculated according to the formula of Yue et al. [21].

### Transesterification reactions

Transesterification was performed in a mixture of 1-butanol and buffer (92.5% 1-butanol, 7.5% buffer (*v*/v)) in a 1 mL final volume. A stock solution of 20 mM MFA dissolved in 1-butanol was prepared. Reactions were run in a thermomixer (Eppendorf, Hambourg, Germany) at 40 °C with shaking at 700 rpm. 1-Butanol containing MFA was preheated for 5 min and the reaction was started by the addition of 75 μL of 1000-fold diluted enzyme, or by the addition of 6 mg dry MPS particles carrying the enzyme and 75 μL buffer. The first 50 μL sample was taken immediately. The next 50 μL sample was taken after 14 h of incubation, which was the time when product became detectable. 50 μL samples were then taken every 1 h until the reaction had progressed for 22 h. Before each sample was taken out, the micro-centrifuge tube was thoroughly vortexed to ensure the mixture was uniform and therefore that the enzyme to reactant ratio was not affected by the sampling. At the enzyme dilution chosen, reactions were linear during the whole course of the experiment which means that the reaction thermodynamic equilibrium was not reached yet. Samples were quenched immediately after removal from the reaction mixture by the addition of glacial acetic acid (30% (*v*/v) final concentration). If not analyzed immediately, the samples were stored at − 20 °C. Quantification of MFA, FA and BFA in the samples was performed using high pressure liquid chromatography (HPLC), as described previously by Thörn et al. [7]. Briefly, samples from transesterification reactions were analyzed using a reversed-phase column (Kinetex 2.6u C18 100A 100 × 4.6 mm (Phenomenex, Torrance, California, USA)) and isocratic elution with methanol:acetic acid:water (70:1:29 v/v). Standards of MFA, FA and BFA in the range 0.1–20 mM were used for identification and quantification.

### Determination of the selectivity ratio during transesterification reactions

The selectivity ratio was defined as the molar ratio between the transesterification product BFA and the hydrolysis product FA. Transesterification reactions were performed as described previously with the following modifications. To assess the effect of the immobilization pH, the reactions were run in 0.2 M phosphate-citrate buffer at pH 7.0. To assess the effect of reaction buffer and pH, reactions were run in three different buffers: 0.2 M phosphate-citrate, 0.1 M MOPS and 0.1 M Bis-Tris. Different pH values were used with each buffer (within their buffering pH range). The influence of pore size was evaluated by performing immobilization at pH 6.5 and the enzymatic reaction at pH 7.0 in 0.2 M phosphate-citrate buffer. The effect of water content was evaluated by varying the buffer: 1-Butanol ratio from 0.1 to 20% (*v*/v) in the reaction system using 0.2 M phosphate-citrate buffer pH 7.0.

### Determination of optimal reaction conditions

Transesterification and hydrolysis reactions were performed as described previously. The optimum conditions were determined by varying the pH of the phosphate-citrate buffer from pH 5.0 to pH 8.0 at 40 °C, or the temperature from 15 to 80 °C at pH 7.0. When studying transesterification reactions the reaction system was a mixture of buffer and 1-butanol (7.5% buffer; 92.5% 1-butanol) and only the pH of the buffer can be controlled.

### Determination of kinetic parameters

Kinetic parameters were investigated at the optimum conditions determined for each of the four reactions studied: hydrolysis and transesterification, with free and immobilized enzyme. Transesterification and hydrolysis reactions were performed as described previously. Substrate concentrations were varied in different intervals for the different reactions: 0.05–2.5 mM for hydrolysis with free or immobilized enzyme; 5–60 mM for transesterification with free enzyme, and 10–200 mM for transesterification with immobilized enzyme. After verifying that all the reactions rates measured were the initial rates, the affinity constant K_m_ and maximal velocity V_m_ were determined by nonlinear regression using the “Enzyme kinetic” module from Sigma-plot (Systat Software Inc., San Jose, USA) which is based on the Michaelis-Menten eq. [22]. The turnover number k_cat_ was subsequently calculated. The product formation was quantified as described previously, using standard curves in the same range as the initial substrate concentration.

### pH and temperature stability

In order to study the pH stability, enzyme was incubated at room temperature for different times (0 to 24 h) in phosphate-citrate buffer in the pH range 5.0–8.0. To study the temperature stability, the enzyme was incubated in phosphate-citrate buffer at pH 7.0 for different times (0 to 24 h) at RT, 40 and 55 °C. After incubation, the hydrolytic activity of the enzyme was assessed at 40 °C for 10 min, as described previously.

### Reusability test

Ten transesterification reaction cycles were performed consecutively using the same immobilized enzyme in MPS with a 9.9 nm pore size, using the experimental setup described previously. Each cycle was performed in 92.5% 1-butanol and 7.5% 0.2 M phosphate-citrate buffer (*v*/v) at 30 °C and 700 rpm. After 48 h the reaction was stopped by centrifugation (15,000 × g, 5 min, RT), the supernatant recovered and stored at − 20 °C for later analysis. Each cycle lasted for 48 h. A new reaction cycle was then initiated by the addition of fresh substrate solution to the immobilized enzyme without any intermediate washing step. The MFA, FA and BFA contents of the supernatants were determined using HPLC as described previously.

## Results

First, immobilization conditions (buffer, immobilization pH, enzyme loading and support pore size) were optimized. Under these conditions, the enzymatic activity was then studied (reaction buffer pH, temperature, water content). The enzyme was then biochemically characterized. Finally the immobilized biocatalyst was evaluated (stability and reusability).

### Optimization of the immobilization parameters

Several factors during the immobilization process can influence enzyme immobilization and enzyme selectivity [23, 24]. Therefore, different buffers were tested at various pH values as well as different enzyme concentration and different pore sizes of the MPS.

### Influence of buffer composition and pH and enzyme concentration on enzyme loading and selectivity

**Fig. 2:**
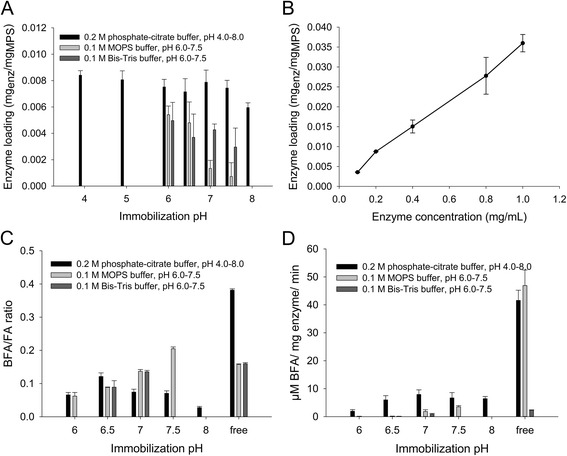
Effect of immobilization pH and of enzyme concentration on loading and enzymatic activity. **a** Effect of immobilization pH on the loading using different buffers. **b** Effect of enzyme concentration on the loading. **c** Influence of the immobilization pH and buffer on the BFA/FA molar ratio. The results are compared to those obtained for the free enzyme at pH 7.0 in the respective buffers. **d** Influence of the immobilization pH on the BFA specific activity. The results are compared to those obtained for the free enzyme at pH 7.0 in the respective buffers. All reactions were in initial velocity. All values are averages of triplicates and the error bars represent one standard deviation

pH of different buffers and their influence on the enzyme loading of the MPS were investigated. The transesterification activity (measured as μM of BFA released per mg of enzyme per min) and selectivity (measured as the BFA/FA molar ratio) of the enzymes immobilized were then assessed. The pore size of the MPS were also varied in order to assess their impact on the loading capacity, activity and selectivity. The enzyme concentration applied to the MPS needed for activity detection was also investigated. Results are presented in Fig. 2 and Table 2.

**Table 2:** Effect of the pore diameter on enzyme loading and the selectivity of immobilized enzymes

Pore size (nm)	Loading (mg_enz_/mg_MPS_)	BFA/FA molar ratio	BFA specific activity (μmol_BFA_/mg_enz_/min)
5.0	0.022 ± 0.003	0.13 ± 0.01	3.92 ± 0.37
7.8	0.021 ± 0.002	0.11 ± 0.02	9.63 ± 1.27
9.9	0.018 ± 0.003	0.12 ± 0.01	8.71 ± 0.34

At the same enzyme concentration of 0.2 mg/mL applied, the best loadings were achieved at different pH values depending on the buffer. While pH 6.0 was the best pH for MOPS buffer and Bis-Tris buffer, the obtained enzyme loading was similar within the pH range 5.0–7.5 for phosphate-citrate buffer. The best enzyme loadings were obtained with phosphate-citrate buffer. It was demonstrated that not only the pH but also the buffer chemical composition influences the enzyme loading (Fig. 2a ).

By applying increasing amounts of enzyme to the MPS, an increasing enzyme loading was observed. The relationship was linear, demonstrating that the maximum enzyme loading capacity of the MPS was not reached in the conditions the assays were performed in the present study (Fig. 2b). The transesterification activity of the samples was evaluated in a mixture of 1-butanol and phosphate-citrate buffer, at pH 7.0. In the defined assay conditions, activity was first detected when enzyme solutions at 0.4 mg/mL were loaded on MPS. No significant differences were observed between the different enzyme loadings neither on the BFA/FA molar ratio nor on the BFA specific activity when concentrations of enzymes ranging from 0.4 to 1 mg/mL were applied (data not shown).

Immobilization pH also influences the enzyme activity and selectivity when the immobilized enzymes are used [24]. Enzymes were immobilized in the different buffers at various pHs using a concentration of 0.4 mg/mL. A similar pattern was observed with this enzyme concentration (data not shown) as the one observed with 0.2 mg/mL (Fig. 2a). The transesterification reaction was then studied in a mixture of 1-butanol and phosphate-citrate buffer at pH 7.0. Transesterification reactions were monitored from the moment the product became detectable (14 h of reaction) until 22 h of reaction. During this time frame, all reactions showed a linear increase of the products which is characteristic of initial rates and therefore the thermodynamic equilibrium was not reached. The influence of immobilization pH on selectivity was investigated by measuring the BFA/FA molar ratio. The performance was assessed by the BFA specific activity. Results are shown in Fig. 2a and d. The main activity of the enzyme was hydrolysis under all conditions, since the best BFA/FA molar ratio obtained was 0.4 for the free enzyme. When using phosphate-citrate buffer, the best BFA/FA ratio was obtained at a pH of 6.5. When using Bis-Tris buffer the FAE showed activity only at immobilization pH of 6.5 and 7.0, and with MOPS buffer an increasing BFA/FA ratio was observed with increasing pH, showing a maximum of 0.2 at pH 7.5. Specific activities showed a clear preference of the enzyme for the phosphate-citrate buffer. Phosphate-citrate buffer at a pH of 6.5 was selected as immobilization buffer for the proceeding experiments, as a compromise between high loading and high selectivity.

### Influence of pore size on enzyme activity

Enzyme loading can also be influenced by the pore size of the particles. For example, if the pores are small, they can limit diffusion of the enzyme inside the pores [20]. Therefore, immobilization was performed in particles with pores of three different diameters, 5, 7.8 and 9.9 nm. The results are presented in Table 2.

Using phosphate-citrate buffer at pH 6.5 as the immobilization buffer resulted in a similar enzyme loading for all three pore diameters tested. The pore size of the MPS did not have any influence on the BFA/FA molar ratio either, but significantly influenced the BFA specific activity. With the smaller pore size (5 nm) a decrease in the BFA specific activity of almost 2.5 fold was observed, compared with the larger pore sizes.

### Optimization of reaction conditions

It has been demonstrated previously that the buffer, pH, and water content of the reaction system can affect the enzyme selectivity in synthetic reactions [24]. More specifically, these parameters can affect the molar ratio between the transesterification product BFA and the hydrolysis product FA. Therefore, we studied the influence of the above mentioned parameters. It is also important to evaluate the optimum temperature and pH when working with enzymes.

### Influence of reaction pH on selectivity

**Fig. 3:**
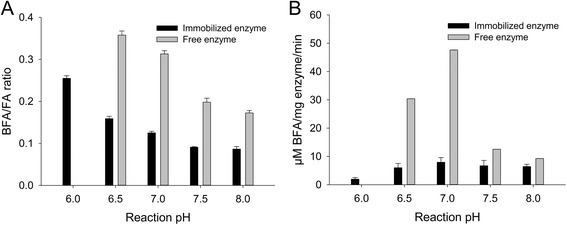
Effect of the reaction pH for free and immobilized enzyme. **a** Effect on the BFA/FA molar ratio. **b** Effect on the BFA specific activity. (Reactions were performed in a 1-butanol (92.5%) phosphate-citrate buffer (7.5%) mixture. The pH of the buffer fraction was varied from 6.0 to 8.0. Immobilization was performed at pH 6.5 and only the reaction pH was varied.) All reactions were in initial velocity. The results are the average of triplicate samples, and the error bars represent one standard deviation

In order to assess only the effect of the reaction pH, all enzymes were immobilized in 0.2 M phosphate-citrate buffer at pH 6.5. The immobilized enzyme was then washed, dried and resuspended in a mixture of 1-butanol and phosphate-citrate buffer, and the pH of the reaction buffer was varied from 6.0 to 8.0. The BFA/FA molar ratio decreased with increasing reaction pH for both free and immobilized enzyme (Fig. 3), indicating that the conditions for hydrolysis were more favorable at higher pH.

### Influence of water content

**Fig. 4:**
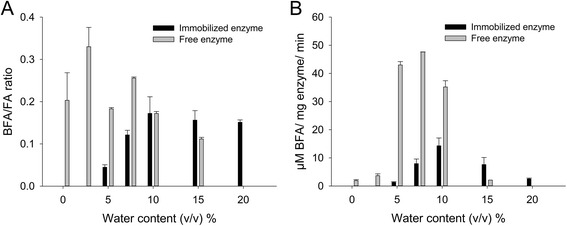
Influence of the water content on the selectivity of free and immobilized enzyme. **a** Effect on the BFA/FA ratio. **b** Effect on the BFA activity. All reactions were in initial velocity. The results are averages of triplicate samples, and the error bars represent one standard deviation

The water content of the reaction system is an important parameter in synthetic reactions. The water content of the reaction system, expressed as the volumetric percentage, influenced the transesterification/hydrolysis molar ratio as shown in Fig. 4.

The immobilized enzyme was not active below 5% water content in the reaction, while above 10% the BFA/FA ratio seemed to stabilize. The free enzyme was, unexpectedly, more tolerant to low water contents; activity being detected with a water content of 0.1% of the reaction volume. The optimum water content for the free enzyme was 2.5%, and for the immobilized enzyme, 10%. Taking the BFA specific activity into account, the best results were obtained at 7.5% and 10% for the free and immobilized enzyme, respectively. A water content as low as possible which still enabled enough activity was needed, therefore 7.5% was chosen for the following experiments.

### Optimum pH and temperature for hydrolysis and transesterification reactions

**Fig. 5:**
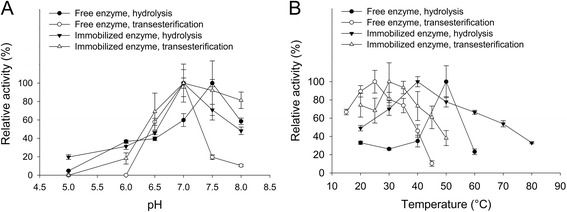
Determination of pH and temperature optima for the four reaction systems studied **a** Optimum pH. Reaction rates measured using phosphate-citrate buffer at pH 5.0–8.0 and 40 °C. **b** Optimum temperature. The temperature was varied from 15 to 80 °C and the reaction was performed using phosphate-citrate buffer at pH 7.0. All reactions were in initial velocity. The results are the average of triplicate samples, and the error bars represent one standard deviation

Following immobilization under the optimal conditions described above, the optimal pH and temperature for hydrolysis and transesterification reactions were determined for both free and immobilized enzyme. The results are shown in Fig. 5.

The pH optima were similar for all reaction systems, pH 7.0, apart from the free enzyme in hydrolysis, which had an optimum at pH 7.5 (Fig. 5a). The latter value differed by one pH unit from the supplier’s data (Megazyme, optimum pH at 6.5 for hydrolysis of ethyl ferulate in 100 mM sodium phosphate buffer). However, since the present experiment was performed using a different substrate and buffer, the values are not directly comparable. Moreover, it has previously been reported that the optimum pH can vary by up to 1 pH unit depending on the reaction catalyzed by the enzyme [25, 26]. An increase of 5 °C in the optimum temperature for transesterification was observed for the immobilized enzyme compared to the free enzyme (Fig. 5b). Immobilization decreased the optimum temperature when the enzyme was studied in hydrolysis; the optima being at 50 °C and 40 °C for free and immobilized enzyme, respectively (Fig. 5b). However, the observed profiles for immobilized enzyme were broader, leading to better relative activities at higher temperatures. Hence, the immobilized enzyme appears to be more stable as it retains more than 60% of its activity after incubation at 60 °C for 10 min, while the free enzyme retains less than 25%. The addition of 1-butanol to the mixture, required for the transesterification reaction, caused a decrease in the temperature optima for both the free and the immobilized enzyme of 25 °C and 10 °C, respectively (Fig. 5b).

### Kinetic parameters of the immobilized biocatalyst

In order to assess the effects of immobilization on the enzyme behavior, the kinetic parameters (V_m_, K_m_ and k_cat_) were determined based on the Michaelis-Menten eq. [22]. It could be argued that the transesterification reaction studied was a two-substrate reaction employing butanol and MFA as substrates. However, since the 1-butanol content of the reaction was not varied during the reaction, and 1-butanol was always present in excess, the kinetics for a single-substrate reaction deemed applicable. Moreover, most of the enzyme kinetics can be estimated by the Michaelis-Menten equation when the concentration of one substrate is kept constant [27].

The affinity constant, K_m_, of the enzyme was negligibly affected by immobilization (Table 3), however, a more than 100-fold decrease in the affinity to MFA was observed when transesterification was compared to hydrolysis. The maximum decrease in the turnover number, k_cat_, of the enzyme upon immobilization was 10-fold. Furthermore, a 100-fold lower turnover number was observed in transesterification than in hydrolysis, for both the free and the immobilized enzyme. The resulting catalytic efficiency, k_cat_/K_m_, of the enzyme was therefore lower when the enzyme was immobilized than its free counterpart: up to 11-fold lower. A dramatic decrease was observed when the transesterification reaction was compared to the hydrolysis reaction with a catalytic efficiency 20,000 times and 43,000 times lower for the free and immobilized enzyme, respectively.

**Table 3:** Kinetic parameters for the four reactions systems studied

	K_m_^a^ (mM)	k_cat_^a^ (s^− 1^)	k_cat_/K_m_^a^ (s^− 1^ M^− 1^)
Free enzyme - hydrolysis	0.43 ± 0.07	31.4 ± 2.20	7.29.10^4^ ± 1.7.10^4^
Free enzyme - transesterification	36.0 ± 14.4	0.14 ± 0.03	3.79 ± 2.33
Immobilized enzyme - hydrolysis	0.43 ± 0.06	6.47 ± 0.34	1.5.10^4^ ± 0.3.10^4^
Immobilized enzyme - transesterification	30.9 ± 7.30	0.01 ± 1.10^− 3^	0.35 ± 0.11

^a^Data are presented as mean ± standard deviation

### Evaluation of the stability and reusability of the immobilized biocatalyst

The effect of immobilization on the long-term stability of the FAE at different pH and temperatures was also investigated. Since the pH optima were similar for the free and immobilized enzyme (Fig. 5a) similar behavior could be expected in long term stability (24 h). However, the free enzyme was more stable at all pH values investigated than the immobilized enzyme (Additional file 1 Figure S2). The effect of temperature during a 24 h incubation showed a similar pattern with only the exception of a slight beneficial effect of immobilization at 55 °C (Additional file 1 Figure S3).

**Fig. 6:**
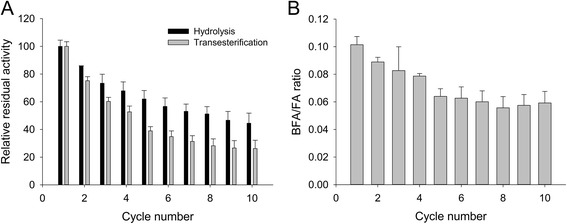
Evaluation of the reusability of the immobilized enzyme over 10 cycles of 48 h each. **a** Relative residual hydrolytic and transesterification activities. **b** Evolution of the BFA/FA ratio during the experiment. All reactions were in initial velocity. Data are averages of triplicates. Error bars represent one standard deviation

Despite the fact that the study on the kinetic parameters showed a decrease in the catalytic efficiency upon immobilization (Table 3), immobilizing an enzyme may still be advantageous in terms of the reusability of the biocatalyst. Therefore, the reusability of the immobilized enzyme was assessed over ten 48 h cycles. During the 20 days of this study, a decrease was observed in both hydrolytic and transesterification activities (Fig. 6), which could have been due to enzyme denaturation or leakage. The transesterification activity decreased more rapidly and, therefore, the enzyme selectivity for transesterification decreased over time. During the last cycle the immobilized enzyme had retained 26% and 44% of its transesterification and hydrolysis relative activities respectively.

## Discussion

As enzyme immobilization so far relies mostly on trial and error, getting insight of the behavior of different enzymes is of utmost importance. We therefore investigated the influence of different parameters on the immobilization process and determined the optimum reaction conditions. In addition, optimization also had to be performed to enhance the synthetic transesterification activity of the enzyme and decrease its natural hydrolytic activity. The kinetic parameters were determined under the optimum conditions in order to study the effect of immobilization. The stability and reusability of the immobilized biocatalyst were also investigated.

### Immobilization conditions

When varying the immobilization pH, the differences in loading patterns (Fig. 2a) observed between the three buffers demonstrate that not only the pH, but also the chemical composition of the buffer, must be taken into account when performing immobilization. pH affects the enzyme surface charge and therefore the electrostatic interactions between the enzyme and the support [23]. The chemical composition of the buffer can also influence enzyme conformation and in turn change the enzyme-support interactions. For instance, MOPS buffer has been shown to interact with the catalytic site of a FAE from *Fusarium oxysporum*: FoFaeC, in a molecular docking study [24]. Influence of the pH of the immobilization buffer on the enzyme loading [24] and selectivity [7], has been investigated previously for FoFaeC, resulting in an increase of selectivity for transesterification. In another study, using four different FAEs from the thermophilic fungus *Myceliophtora thermophila* C1 and 10.1 nm pore size SBA-15, the selectivity towards transesterification was decreased for three out of four enzymes [28]. In the present study, using a commercial FAE, immobilization led to a decreased selectivity for transesterification (Fig. 2c). Our findings therefore confirm the hypothesis that the selectivity of an enzyme can be changed by immobilization.

The best loading obtained in the present study (0.036 mg ⋅ mg^− 1^) (Fig. 2b) was in the same range as the one obtained for a horseradish peroxidase (lower than 0.03 mg ⋅ mg^− 1^) [29]. However, it was lower than those achieved on SBA-15 for some other enzymes, which ranged from 0.044 to 0.185 mg ⋅ mg^− 1^ for a lipase, a glucose oxidase and papain [8, 9]. Low loading can be explained by the low enzyme concentration used in the present study. This was further exemplified by the loading capacity experiment presented in Fig. 2b where it was demonstrated that the maximum loading was not reached.

When looking at the immobilization in different pore size materials (Table 2), no difference in loading was observed. This finding supports the hypothesis that the FAE, (which diameter was estimated to be around 4 nm based on its molecular weight of 29 kDa and assuming that it is perfectly spherical) can enter the pores equally well in all the materials investigated. A lower specific activity was though observed for the material with the smaller pore size. This could be due to limitations on the diffusion of the substrate in the 5 nm pores since the estimated diameter of the enzyme is around 4 nm. Another possible explanation is increased rigidity of the enzyme due to more attachment points with the support, since the size of the enzyme is expected to be only slightly smaller than the size of the pores. There may also be some loss of activity due to enzyme orientation with its active site facing the walls, thus preventing the substrate from entering the enzyme active site.

Changing the pH of the reaction mixture may result in a change in the charge distribution of the amino acid residues of the enzyme, inducing slight modifications in their conformation, as well as in the way in which the enzyme interacts with its immobilization support [23]. Such changes in enzyme conformation could explain the shift in selectivity observed when changing the reaction pH (Fig. 3). The best selectivity ratio was obtained at a reaction pH of 6.5 and 6.0 for the free and immobilized enzyme, respectively (Fig. 3a). However, a low reaction pH is not necessarily suitable in an industrial application as the specific activity of the enzyme is not optimal at low pH as demonstrated by the specific activities (Fig. 3b) which in this case were lower at pH 6.0 than at pH 7.0. A compromise must thus be made between enzyme selectivity and enzyme specific activity.

### Immobilization did not lead to changed substrate affinity

A biochemical characterization of the enzyme was performed in four different reaction systems: (i) free enzyme – hydrolysis reaction, (ii) free enzyme – transesterifications reaction, (iii) immobilized enzyme – hydrolysis reaction and (iv) immobilized enzyme – transesterification reaction. The optimum pH and temperature of the enzyme were investigated first. The free enzyme optimum pH was found to be 7.5 (Fig. 5a). When looking at the immobilized enzyme, optimum pH decreased to 7.0. A shift in pH optimum upon immobilization has been reported previously for another FAE [7], and could be explained by the different microenvironment inside the pores of mesoporous materials [2, 4].

The optimum temperature of the free and immobilized enzymes studied in transesterification were decreased compared with the enzymes studied in hydrolysis (Fig. 5b). When the enzyme was free, a 15 °C decrease was observed. When it was immobilized the decrease was of 10 °C. This points to the fact that 1-butanol has a negative effect on the thermostability of the enzyme which is consistent with the fact that enzymes usually exhibit lower activities in organic solvents than in aqueous systems due to partial or complete denaturation of the enzyme [30]. The observed decrease in the temperature optima for the immobilized enzyme was less dramatic. Together with the broader profiles observed, this may indicate that enzyme immobilization had a thermo-stabilizing effect, and a possible sheltering effect of the MPS pore system. The stabilizing effect of immobilization has been reported for other enzymes, and could be due to reduced flexibility of the enzyme due to attachment to the support, thus preventing thermal denaturation and reducing the negative effect of solvents such as 1-butanol which are known to destabilize protein structures [11].

Upon immobilization, K_m_ did not change, however, k_cat_ was decreased (Table 3); suggesting that there were no limitations on diffusion or mass transfer with the pore diameter used in these experiments (9.9 nm). It can therefore be concluded that immobilization reduced the reaction rate of the enzyme. Changes in kinetic parameters have been observed previously in other immobilized enzymes. A tendency towards positive effects has been seen in the case of lipases, with almost a doubling of the maximal velocity, V_m_ [31, 32], while negative effects have been reported for 2,3-dihydroxybiphenyl 1,2-dioxygenase immobilized on SBA-15, for which K_m_ increased 2.8 times and k_cat_ decreased nearly 4-fold [33].

### Stability and reusability

It was clear that immobilization did not have a long-term stabilizing effect. It is known that physical adsorption is not the best strategy for improving the stability of an enzyme [2]. However, for this enzyme it was observed no improvement in long-term stability due to immobilization but also minimal effects of immobilization on the optimal reaction conditions as well as minimal effects of immobilization on the enzyme selectivity. Taken together, these results suggest a naturally high rigidity of the enzyme, at least in the area around its active site [2].

Looking into the reusability of the immobilized biocatalyst in transesterification, over ten cycles of 48 h, a shift in selectivity towards hydrolysis was observed (Fig. 6). Despite that, the immobilized enzymes retained 26% and 44% of their transesterification and hydrolysis relative activities, respectively, at the end of the experiment. Considering the harsh conditions (92.5% butanol) in which the enzymes were put, the retained activities can be considered good. However, it is likely that no gain in terms of the total amount of BFA produced would be obtained by immobilizing this enzyme on this type of support, since the transesterification activity of the free enzyme was higher than the one of the immobilized enzyme (Fig. 3b). In the present study, enzymes immobilized on MPS were dried before being used in transesterification reactions. One way to improve the BFA yield of immobilized enzymes might be to replace the drying step by solvent rinsing as it has been shown to improve the productivity of a FAE from *Myceliophtora thermophila* C1 from 3 to 5 mmol_BFA_/g_enz_/h [28].

Because of its inherently good stability, this enzyme has some potential to become a good immobilized biocatalyst. But more work is needed to find the appropriate support and/or immobilization technique. It has also been shown that the solvent-buffer system used influences the reaction rate [34], which could also offer means of improving the enzyme performance. Among the parameters investigated, the water content and the pH of the buffer, during both immobilization and reaction, proved to be critical in improving the transesterification ratio.

## Conclusions

Immobilizing the FAE: E-FAERU resulted in a lowered transesterification efficiency. In earlier studies, changes of selectivity have also been observed upon immobilization of FAEs on SBA-15. Overall, regarding the selectivity of E-FAERU, the hydrolysis reaction was preferred by the enzyme regardless if it was free or immobilized. The long-term stability of the immobilized FAE was improved at 55 °C, whereas no improvement was observed under other conditions. However, improvements in short-term stability of more than 2-fold were obtained when defining the temperature profile. Under optimized immobilization and reaction conditions it was demonstrated that immobilization did not affect the K_m_ of the studied FAE. However, the turnover number, k_cat_, decreased, leading to a decrease in the overall catalytic efficiency. The findings of this study have also underlined the importance of the choice of pH and buffer during the immobilization process. Overall, this demonstrates that the results are enzyme-specific, and cannot be regarded as reflecting the general behavior of FAEs in MPS.

## Additional file

Additional file 1: TEM image of the used MPS and stability evaluation of the FAE. **Figure S1.** TEM image of the calcined SBA-15 mesoporous silica material with a 9.9 nm pore size used in this study. **Figure S2.** pH stability of the enzyme during 24 h. Four pH values were assessed in 0.2 M phosphate-citrate buffer at room temperature. (A) Free enzyme. (B) Immobilized enzyme. Data are averages of triplicates. Error bars represent one standard deviation. **Figure S3.** Temperature stability of the enzyme during 24 h. Three temperatures were tested in 0.2 M phosphate-citrate buffer pH 6.5. (A) Free enzyme. (B) Immobilized enzyme. Data are averages of triplicates. Error bars represent one standard deviation. (DOCX 175 kb)

## Abbreviations

BFA: Butyl ferulate
Bis-Tris: bis(2-hydroxyethyl)amino-tris(hydroxymethyl)methane
BSA: Bovine serum albumin
FA: Ferulic acid
FAE: Feruloyl esterase
HPLC: High pressure liquid chromatography
MFA: Methyl ferulate
MOPS: 3-(N-morpholino)propanesulfonic acid
MPS: Mesoporous silica particles
RT: Room temperature
SBA-15: Santa Barbara Amorphous Type 15
