# Supplementary material for: Feruloyl esterase immobilization in mesoporous silica particles and characterization in hydrolysis and transesterification
Source: BMC Biochem. 2018 Feb 2;19:1. doi: 10.1186/s12858-018-0091-y (PMC5795792; doi:10.1186/s12858-018-0091-y)
Supplement: Additional file 1: — TEM image of the used MPS and stability evaluation of the FAE. Figure S1. TEM image of the calcined SBA-15 mesoporous silica material with a 9.9 nm pore size used in this study. Figure S2. pH stability of the enzyme during 24 h. Four pH values were assessed in 0.2 M phosphate-citrate buffer at room temperature. (A) Free enzyme. (B) Immobilized enzyme. Data are averages of triplicates. Error bars represent one standard deviation. Figure S3. Temperature stability of the enzyme during 24 h. Three temperatures were tested in 0.2 M phosphate-citrate buffer pH 6.5. (A) Free enzyme. (B) Immobilized enzyme. Data are averages of triplicates. Error bars represent one standard deviation. (DOCX 175 kb) [file 12858_2018_91_MOESM1_ESM.docx]

# BMC Biochemistry

**Supplementary information**

# Feruloyl esterase immobilization in mesoporous silica particles and characterization in hydrolysis and transesterification.

Cyrielle Bonzom^a^, Laura Schild^a^, Hanna Gustafsson^b^^[[1]](#footnote-1)^ and Lisbeth Olsson^a^*

^a^Chalmers University of Technology, Department of Biology and Biological Engineering, Industrial Biotechnology Division, SE-412 96 Gothenburg, Sweden

^b^Chalmers University of Technology, Department of Chemical and Biological Engineering, Applied Surface Chemistry, SE-412 96 Gothenburg, Sweden

*Corresponding author

E-mail: [lisbeth.olsson@chalmers.se](mailto:lisbeth.olsson@chalmers.se)

Phone: +46 31 772 3805

Fax: +46 31 772 3801


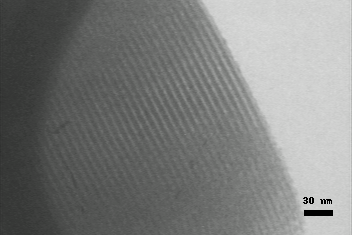


Figure S1 TEM image of the calcined SBA-15 mesoporous silica material with a 9.9 nm pore size used in this study.





Figure S2 pH stability of the enzyme during 24hours. Four pH values were assessed in 0.2M phosphate-citrate buffer at room temperature. (A) Free enzyme. (B) Immobilized enzyme. Data are averages of triplicates. Error bars represent one standard deviation.





**Figure S3** Temperature stability of the enzyme during 24hours. Three temperatures were tested in 0.2M phosphate-citrate buffer pH6.5. (A) Free enzyme. (B) Immobilized enzyme. Data are averages of triplicates. Error bars represent one standard deviation.

1. Present address: Chalmers University of Technology, Department of Applied Physics, Biological Physics Division, SE-412 96 Gothenburg, Sweden [↑](#footnote-ref-1)
